# Cardiometabolic risk profile among children with migrant parents and role of parental education: the IDEFICS/I.Family cohort

**DOI:** 10.1038/s41366-023-01359-5

**Published:** 2023-09-01

**Authors:** Anna Lindblad, Florence Samkange-Zeeb, Stefaan de Henauw, Antonia Solea, Toomas Veidebaum, Fabio Lauria, Luis A. Moreno, Isabel Iguacel, Dénes Molnár, Wolfgang Ahrens, Volker Winkler, Lauren Lissner, Kirsten Mehlig

**Affiliations:** 1 Epidemiology of Transition, Heidelberg Institute of Global Health, Heidelberg University Hospital, Heidelberg, Germany; 2 Leibniz Institute for Prevention Research and Epidemiology – BIPS, Bremen, Germany; 3 Department of Public Health and Primary Care, Faculty of Medicine and Health Sciences, Ghent University, Ghent, Belgium; 4 Research and Education Institute of Child Health, Strovolos, Cyprus; 5 Department of Chronic Diseases, National Institute for Health Development, Tallinn, Estonia; 6 Institute of Food Sciences, National Research Council, Avellino, Italy; 7 GENUD (Growth, Exercise, Nutrition and Development) Research Group, University of Zaragoza, Zaragoza, Spain; 8 Centro de Investigación Biomédica en Red de Fisiopatología de la Obesidad y Nutrición (CIBERObn), Instituto de Salud Carlos III, Madrid, Spain; 9 Instituto Agroalimentario de Aragón (IA2), Zaragoza, Spain; 10 Instituto de Investigación Sanitaria Aragón (IIS Aragón), Zaragoza, Spain; 11 Department of Pediatrics, Medical School, University of Pécs, Pécs, Hungary; 12 School of Public Health and Community Medicine, Institute of Medicine, Sahlgrenska Academy, University of Gothenburg, Gothenburg, Sweden

**Keywords:** Risk factors, Obesity

## Abstract

**Background and aims:**

Evidence shows that migrant children have a higher risk of developing obesity than those with native parents. We aimed to investigate the association between parental migration background and cardiometabolic health in children and adolescents in Europe.

**Methods and results:**

We included 8745 children aged 2–17 from the second follow-up of the European IDEFICS/I.Family cohort. Linear regression models were used to investigate the association between parental migration background (one or two migrant parent(s) vs native parents) and body mass index (BMI), metabolic syndrome (MetS) score and its individual components. Outcome variables were parametrized as age and sex-specific z-scores. We adjusted for age, sex, country, and parental education, and additionally for parental income, lifestyle including dietary factors, and maternal BMI. On average, children with two migrant parents had higher z-scores of BMI (+0.24 standard deviation (SD)) and MetS score (+0.30 SD) compared to those with native parents, whereas no significant differences were seen for children with one migrant parent. Associations were attenuated when controlling for maternal BMI and sports club activity. Parental education modified the associations with BMI and MetS z-scores such that they were more pronounced in children with low parental education.

**Conclusion:**

Children with two migrant parents were at higher risk for adverse cardiometabolic health compared to children with native parents, especially in families with low parental education. These associations were explained by lower physical activity and maternal body weight and encourages early intervention strategies by schools and communities.

## Background

Over recent decades, the prevalence of childhood overweight and obesity have continued to rise in many parts of the world [1], in parallel with increasing migration [2]. Several studies reported that children who immigrated in Western countries tend to have a higher risk of developing overweight and obesity than their native peers [3, 4]. Factors including lifestyle and dietary patterns [5–7], acculturation [8], maternal overweight [9], socioeconomic status [7], and food insecurity [4] have been discussed or found to explain the higher risk of excess body weight among children with migration background. Generally, childhood overweight including obesity causes weight-related health problems such as hypertension and insulin resistance leading to increased morbidity and mortality in adulthood [1, 10]. It is therefore necessary to examine early signs of metabolic disease that go beyond anthropometric measures.

In contrast to the definition of metabolic syndrome (MetS) in adults, there is no generally accepted definition for children [11], mainly due to the lack of reference values that account for the developmental changes during childhood. Because projected prevalences of childhood metabolic syndrome are low Eisenmann advocated the use of scores reflecting the continuum between healthy and unhealthy cardiometabolic profile in children [12]. To account for developmental changes further attempts to define MetS scores in children relied on combinations of sex- and age-specific z-scores for the individual components of the metabolic syndrome [13, 14]. Along these lines Ahrens et al. developed a MetS score based on data from over 18,000 children aged between 2 and 9 years and participating in the European IDEFICS (Identification and prevention of dietary- and lifestyle-induced health effects in children and infants) study [15]. Cross-sectional results from the IDEFICS study showed that children with parental migration background were more likely to be overweight/obese [5], eat more processed food [16], and report longer screen time compared to children with native parents [17]. However, no difference by migration background was found regarding the metabolic syndrome score [18].

The present study aims to take these analyses one step further by examining the association between parental migration background and indicators of cardiometabolic health – including BMI, the metabolic syndrome score and its components – based on the second follow-up of the IDEFICS/I.Family cohort including children and adolescents up to 17 years of age [19]. We hypothesize that the impact of having one or two migrant parents on cardiometabolic health emerges as children become adolescents. Furthermore, we aim to understand which factors besides migration history, e.g. socioeconomic factors, lifestyle and dietary factors, were underlying explanations for excess cardiometabolic risk in children of immigrants in Europe.

## Materials and methods

### Study population

The present study is based on the multicentre IDEFICS/I.Family cohort, which includes children from Belgium, Cyprus, Estonia, Germany, Hungary, Italy, Spain, and Sweden. Detailed information on the design and objectives of the IDEFICS/I.Family studies can be found in Ahrens et al. [19–21]. In brief, the baseline survey 2007/2008 included 16,224 children aged 2–9 years who attended kindergarten or primary school in selected communities with comparable infrastructure and sociodemographic profile in each country. In the first follow-up in 2009/2010, additional 2555 children were recruited. The second follow-up was conducted in 2013/2014 and is referred to as I.Family (“I.” refers to “IDEFICS”). To investigate the influence of family characteristics additional family members were included, among them 2512 siblings aged <18 years. Children and adolescents participating in I.Family were 2.0–17.9 years old, with 37% from baseline, and 41% from first follow-up participating in the second follow-up [21].

The initial dataset from the second follow-up comprised 17,600 participating children and parents. For the present project, we excluded observations from parents and other adults (age ≥18 years, *N* = 7983) and observations with missing values for BMI (*N* = 36), parental education (*N* = 83) or parental migration background (*N* = 753). The final analytic sample included 8745 children and adolescents within the age span 2–17 years. Furthermore, non-fasted blood samples were excluded in the analyses of blood biomarkers (*N* = 983).

### Methods

Data on sociodemographic variables and lifestyle were collected by questionnaires that were completed by the parents with the exception that adolescents aged ≥12 years reported on lifestyle and diet by themselves. Anthropometry and blood pressure was measured at physical examinations, and fasting blood samples were taken to measure lipids, insulin and glucose. The main exposure was parental migration background that was reported by parents at baseline or first follow-up. Participating children were divided into three exposure groups based on the number of migrant parents, i.e. none, one, or two parent(s) born in a country other than the country of residence. Outcome variables were z-scores for body mass index (BMI), and for a metabolic syndrome score (MetS score) and its components, i.e. waist circumference (WC), systolic and diastolic blood pressure (SBP, DBP), high-density lipoprotein cholesterol (HDL-C), triglycerides (TG), and the homoeostasis model assessment for insulin resistance (HOMA-IR). The BMI z-score was standardized using an external population based on Cole et al. [22], to allow for comparisons with other studies. All other variables were internally standardized into sex and age-specific z-scores [15]. The MetS score was defined as the sum of z-scores for HOMA-IR, WC, mean of z-scores of DBP and SPB, and mean of HDL-C (multiplied with -1 due to inverse relation) and TG, with higher values indicating higher metabolic risk. In order to allow for comparison of associations across different endpoints, we calculated age- and sex-specific z-scores for the MetS score as well [15]. The parents’ highest level of education was classified according to the International Standard Classification of Education (ISCED) 2011 and divided into two categories: low for primary/secondary/post-secondary non-tertiary education (ISCED 0–4), and high for any tertiary education (ISCED 5–8) [23]. Parental income was based on nine country-specific levels in relation to the average country-specific monthly net household income, and dichotomized into low (level 1-3) and high (level 4-9) income level [24]. Single parent household was defined as living with one parent/adult. Maternal BMI was calculated from mothers’ measured height and weight.

To identify potential confounders for the association between parental migration background and health indicators, we examined the following variables: sports club activity as a proxy for physical activity (hours per week), screen time as a proxy for sedentary behaviour (hours per week using TV or PC), consumption of fast-food (frequency per day), consumption of fruit and vegetables (frequency per day), healthy diet score (based on adherence to nutrition guidelines on consumption of fruit and vegetables, fish, whole grains, sugar, and fat quantity [25]), and well-being (based on the dimensions emotional well-being, self-esteem, relations with family and peers, based on a generic quality of life instrument for children called the KINDL-R questionnaire [26]). To further characterize the countries of origin, we used the Human Development Index (HDI), which describes a country’s developmental status based on life expectancy, education, and income per capita [27]. HDI per family was calculated as the mean HDI of both parents’ countries of origin.

### Statistical analyses

Study characteristics were given in terms of mean values (standard deviation (SD)) and numbers (column percent) for continuous and categorical variables, respectively. Differences by parental migration background were tested based on Kruskal Wallis (continuous variables) and Chi-square tests (categorical variables). We used linear regression models to investigate the associations between parental migration background and z-scores for BMI and MetS as well as its components, including random effects to account for correlations between children within families nested within the communities in each country. To examine the individual contribution of having one or two migrant parents compared to native parents in relation to health outcomes, parental migration background was treated as a categorical exposure with two native parents as the reference group. Two types of regression models were performed for all outcomes. In model 1, associations with parental migration background were adjusted for sex, age, country of residence, and parental education level. Sex and age were included as covariates because a) the analytic sample differed from the sample used for standardization, and b) an external reference was used for BMI z-score. In model 2, additional adjustments were made for parental income level, maternal BMI, lifestyle and dietary habits, i.e. factors that differed by parental migration background in univariate analyses. The results of linear regression models were given in terms of beta-coefficients with confidence intervals (CI). Because the outcome variables are z-scores, the units of beta-coefficients are given by the SD of the outome variable per unit of the predicting variable. Interaction analyses for migration background and the variables sex, age group (dichotomized at age 12), country of residence, and parental education were carried out by including the respective product terms in model 1. Interaction results were presented as stratum-specific associations, and an overall p-value for interaction was derived from comparing a model with and without interaction terms. The following sensitivity analyses were carried out: first, we performed analyses for model 1 in the identical subsample of children as in model 2 to compare associations. Second, we examined whether previous participation in an early intervention programme as part of the IDEFICS study influenced the results [20]. Third, in the Cypriot sample we re-defined parents born in Greece as natives, motivated by their closeness in culture and language. Statistical analyses were performed using Stata IC, version 16 (Statacorp, College Station, TX, USA) and MATLAB (R2016b; The MathWorks, Inc.). To account for multiple comparisons, the statistical significance level was set to 0.01. Results were given in terms of beta values with 99% CI.

## Results

### Descriptive results

Table 1 summarizes the characteristics of the study population by categories of parental migration background including descriptive results for the main outcome variables. The age span was 2.0–17.9 years, the median age 11.2 years, and 49% of the participants were females. Of all children with two migrant parents, about half lived in Germany and 25% in Cyprus, whereas only 1% lived in Estonia, Belgium, or Hungary. The proportion of children with high parental education or high parental income was largest among children with two native parents and smallest among children with two migrant parents. There was no difference in the number of children living in single parent households with regard to migration background. The average number of children per family did not differ by parental migration background. Mean maternal BMI was highest among children with two migrant parents. Regarding lifestyle and dietary factors, children with two migrant parents spent less hours in sports club activities per week but consumed more fruit and vegetables than the other children. No differences were observed regarding fast-food intake, healthy diet score, screen time, or well-being. The population comprised 5596 families, with an average number of children per family of 1.6. More than half of the families participated with only one child (55%). Overall, there was a trend of lower HDI values for country of origin in families with one or two migrant parents, respectively, compared to families with two native parents (Table S1).

**Table 1:** Description of the study population including cardiometabolic health indicators and lifestyle/dietary factors stratified by parental migration background^a^.

Variables	Total no. of children	Native parents (*N* = 7350)	One migrant parent (*N* = 888)	Two migrant parents (*N* = 507)
Age (years) (mean, (SD))	8745	11.0 (2.8)	10.8 (2.9)	11.2 (2.9)
Female sex (*N*, (%))	8745	3617 (49)	428 (48)	263 (52)
Country of residence (*N*, (%))				
Sweden	820	**696 (9)**	**76 (9)**	**48 (9)**
Estonia	1263	**1210 (16)**	**47 (5)**	**6 (1)**
Hungary	1063	**1023 (14)**	**34 (4)**	**6 (1)**
Germany	1205	**837 (11)**	**107 (12)**	**261 (51)**
Belgium	381	**362 (5)**	**14 (2)**	**5 (1)**
Spain	548	**500 (7)**	**22 (2)**	**26 (5)**
Italy	1426	**1164 (16)**	**233 (26)**	**29 (6)**
Cyprus	2039	**1558 (21)**	**355 (40)**	**126 (25)**
High parental education (*N*, (%))	8745	**3877 (53)**	**441 (50)**	**174 (34)**
High parental income (*N*, (%))	7152	**4526 (74)**	**401 (57)**	**157 (49)**
Single parent household (*N*, (%))	7492	613 (10)	90 (11)	51 (11)
Number of children/family (mean, (SD))	8745	**1.6 (0.7)**	**1.7 (0.7)**	**1.6 (0.7)**
Health outcomes (mean, (SD))				
BMI z-score (Cole 2012)	8745	**0.50 (1.14)**	**0.73 (1.15)**	**0.69 (1.18)**
MetS z-score	3522	−0.16 (0.99)	−0.11 (1.11)	0.01 (0.96)
WC z-score	8279	**0.45 (1.48)**	**0.65 (1.55)**	**0.57 (1.51)**
SBP z-score	7976	0.03 (1.01)	0.08 (1.00)	0.06 (1.01)
DBP z-score	7975	−0.00 (0.99)	−0.11 (0.96)	−0.06 (0.98)
HDL-C z-score	4943	**−0.02 (1.02)**	**−0.15 (1.07)**	**−0.16 (1.04)**
TG z-score	5031	**−0.04 (0.99)**	**−0.00 (1.12)**	**0.26 (0.87)**
HOMA-IR z-score	3556	**0.17 (1.19)**	**0.26 (1.23)**	**0.49 (1.05)**
Maternal BMI (kg/m^2^) (mean, (SD))	5887	**25.6 (5.2)**	**26.5 (6.2)**	**26.7 (5.2)**
Lifestyle/dietary factors (mean, (SD))				
Fruit/vegetables intake per day	7486	**2.9 (2.3)**	**3.2 (2.8)**	**3.5 (2.7)**
Fast-food intake per day	8005	1.2 (1.1)	1.3 (1.1)	1.2 (1.1)
Healthy diet score	7911	18.1 (7.4)	18.1 (7.6)	18.1 (8.2)
Sports club activity (hours/week)	5079	**3.7 (2.5)**	**3.6 (2.8)**	**3.1 (1.8)**
Well-being score	7703	39.4 (5.3)	39.5 (5.1)	39.7 (5.2)
Screen time (hours/week)	7849	16.2 (10.4)	16.7 (10.8)	16.5 (11.5)

Results indicated in bold are significant at 0.01 significance level.

*N* number of observations, *BMI* body mass index, *MetS* metabolic syndrome score, *WC* waist circumference, *SBP* systolic blood pressure, *DBP* diastolic blood pressure, *HDL-C* high-density lipoprotein cholesterol, *TG* triglycerides, *HOMA-IR* homoeostasis model assessment for insulin resistance.

^a^Test for differences by parental migration background: Kruskal Wallis (continuous variables), Chi-square test (categorical variables).

### Associations between parental migration background and cardiometabolic health factors

Table 2 summarizes the results for associations between parental migration background and health outcomes. When controlling for age, sex, country of residence, and parental education level (model 1), there was a positive association for BMI z-score (+0.24 SD) and MetS z-score (+0.30 SD) for children with two migrant parents in comparison to children with native parents. Regarding the components of the MetS score, there were positive associations with WC, TG, and HOMA-IR, but not with SBP, DBP or HDL-C. It is worth mentioning that the association between two vs. no migrant parents and TG z-score showed the largest effect size (+0.35 SD). In model 2, we further adjusted for variables that differed by parental migration background in univariate analyses, i.e. parental income, maternal BMI, daily fruit and vegetable intake, and sports club activity (Table 1). Results from model 2 showed that all associations with parental migration background were attenuated and no longer significant, and explained by confounding with maternal BMI and sports club activity. Specifically, maternal BMI in units of kg/m^2^ was positively associated with z-scores for BMI, 0.06 (0.05, 0.07) SD/kg/m^2^, and MetS score, 0.03 (0.02–0.05) SD/kg/m^2^, and with z-scores for WC, HOMA-IR, as well as lower z-scores of HDL-cholesterol (not shown). Sports club activity (hours/week) was associated with lower z-scores of MetS, −0.04 (−0.07, −0.02) SD/hours/week, and also with WC, SBP, DBP, TG and HOMA-IR (not shown). No significant association between parental migration background and any health outcome was observed for children with one migrant parent in comparison to those with native parents (Table 2). Higher parental income per se was associated with lower values of BMI z-score when adjusted for age, sex, and country, but not significantly so when further adjusted for parental education (not shown).

**Table 2:** Associations between parental migration background and cardiometabolic health indicators by two sets of linear regression models^a^.

		One migrant parent vs native parents		Two migrant parents vs native parents	
	*N*	Coefficient	99% CI	Coefficient	99% CI
BMI z-score					
Model 1	8745	0.074	−0.039, 0.188	**0.240**	**0.090, 0.391**
Model 2	3061	0.050	−0.133, 0.233	0.150	−0.159, 0.459
MetS z-score					
Model 1	3522	−0.008	−0.150, 0.135	**0.295**	**0.095, 0.500**
Model 2	1371	−0.015	−0.250, 0.221	0.090	−0.274, 0.455
WC z-score					
Model 1	8279	0.056	−0.098, 0.211	**0.294**	**0.087, 0.501**
Model 2	2965	−0.029	−0.277, 0.219	0.060	−0.356, 0.476
SBP z-score					
Model 1	7976	0.101	−0.005, 0.206	0.135	−0.008, 0.279
Model 2	2918	0.175	−0.002, 0.352	0.282	−0.018, 0.583
DBP z-score					
Model 1	7975	0.022	−0.077, 0.121	0.120	−0.016, 0.255
Model 2	2917	0.046	−0.126, 0.218	0.241	−0.051, 0.533
HDL-C z-score					
Model 1	4943	−0.014	−0.146, 0.118	−0.161	−0.344, 0.022
Model 2	1915	0.028	−0.195, 0.251	0.179	−0.178, 0.536
TG z-score					
Model 1	5031	0.026	−0.101, 0.153	**0.350**	**0.173, 0.528**
Model 2	1948	0.038	−0.173, 0.252	0.143	−0.200, 0.486
HOMA-IR z-score					
Model 1	3556	0.029	−0.144, 0.201	**0.256**	**0.015, 0.496**
Model 2	1373	0.185	−0.126, 0.496	0.094	−0.387, 0.575

Results indicated in bold are significant at 0.01 significance level.

*N* Number of observations, *CI* confidence intervals, *BMI* body mass index, *MetS score* metabolic syndrome score, *WC* waist circumference, *SBP* systolic blood pressure, *DBP* diastolic blood pressure, *HDL-C* high-density lipoprotein cholesterol, *TG* triglycerides, *HOMA-IR* homoeostasis model assessment for insulin resistance.

^a^Model 1: adjusted for sex, age, country of residence, parental education; Model 2: further adjusted for parental income, maternal BMI, sports club activity, fruit/vegetable intake.

**Fig. 1:**
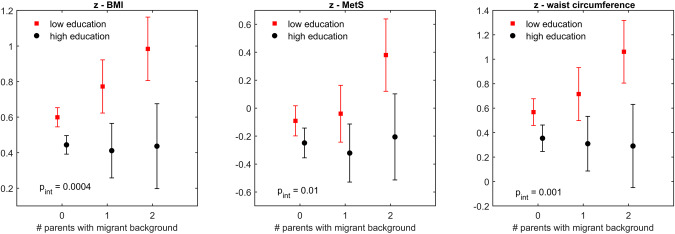
Associations between parental migration background and z-scores for BMI, metabolic syndrome score and waist circumference by parental education. ^a^Marginal mean values adjusted for sex, age, country of residence as well as parental education and migration background including their interaction; *p*-value for interaction between parental migration background and parental education (*p*_int_).

Interaction analyses showed that associations with migration background were substantially stronger in children with low parental education than in children with high parental education, but significantly so only for z-scores of BMI, MetS score, and WC (Table S2). For these z-scores, the contrast between children with two vs. no migrant parents was significantly larger among children with low parental education than among children with high parental education. Among children with low parental education, we also observed a significant association for one versus no migrant parent regarding BMI z-score, with an effect size that was significantly smaller than for two versus no migrant parents, indicating a dose-response relationship between number of migrant parents and children’s BMI z-score. Figure 1 shows that the mean values for z-scores for BMI, MetS score, and WC increased gradually with number of migrant parents among children with low parental education. In contrast, no differences were seen by parental migration background in families with high parental education.

### Country-specific results and sensitivity analyses

Country-specific estimates for associations between parental migration background and z-scores of BMI and MetS score were consistent with the overall results (Table S3). When evaluating model 1 in the same dataset as model 2 (Table S4), the associations between migration background and all outcome variables were attenuated and not significantly different from the results of model 2. Results presented in Table 2 remained unchanged when controlling for participation in the IDEFICS intervention programme, as well as when immigrants from Greece were re-classified as natives (not shown).

## Discussion

To our knowledge, this is the first study to investigate the association between parental migration background and cardiometabolic health beyond weight measures in a large sample of European children and adolescents. Based on sex- and age-specific z-scores for cardiometabolic indicators we showed that children and adolescents with two migrant parents had higher values for BMI and MetS z-scores as well as for WC, TG and HOMA-IR z-scores compared to their peers with two native parents. Effect sizes were of the same order of magnitude for BMI z-score as for metabolic indicators, with a maximum for triglycerides, reinforcing the importance to look at health indicators other than BMI. Children with one migrant parent did not differ significantly in indicators of cardiometabolic health from children with native parents. We observed that parental education strongly modified the associations between parental migration background and cardiometabolic indicators, such that most associations were restricted to children with low parental education. Furthermore, higher maternal BMI and lower physical activity were strongly associated with all cardiometabolic indicators including BMI z-score. Sensitivity analyses comparing adjusted and unadjusted associations in the subsample with confounder information showed that maternal BMI and sports club activity explained only part of the associations between parental migration background and all outcomes.

Higher levels of adverse cardiometabolic health outcomes among children with two migrant parents compared to children with one migrant parent indicate an important difference between the two groups. Previous studies have observed that children with two-parent migration background have higher odds of being overweight and having lower physical activity than peers with one-parent migration background [6, 7]. The better health in children with one migrant parent may be due to the support by the native parent who speaks the local language, knows the culture, and has a social network that may facilitate the integration of the other parent and the entire family. Another explanation could be that the reasons for migration as well as the living conditions before, during and after migration differ between these groups, affecting both health and integration prospects of the family. This is supported by the observation that the mean HDI of parental country of origin was generally lower than the HDI for the country of residence, with a larger contrast between families with two migrant parents than those with one migrant parent. In the German survey sample most migrant parents came from Turkey, Eastern Europe and the former Soviet Union, which is in accordance with the distribution of migrant groups in the total German population [28, 29]. Our results are in line with previous German studies reporting that children of immigrants are more likely to be overweight than children of native-born parents, even after adjustment for socioeconomic status [30, 31]. In contrast, almost half of the Italian parents with migration background were born in Switzerland or Germany, i.e. countries with higher HDI than Italy. It is possible that these parents are children to Italian emigrants, who decided to return to Italy. These families can be expected to integrate well into the local society, which might explain the lack of associations between parental migration background and cardiometabolic health in the Italian survey sample.

Lower socioeconomic status has been associated with childhood overweight and obesity in native as well as migrant families [7, 32]. Different socioeconomic indicators can have both independent and combined effects on risk of childhood obesity [32], but there is evidence that the disparities in overweight and obesity by migration background are prevailing after adjusting for socioeconomic factors [33]. Our results showed protective effects of higher parental education on all indicators of metabolic health investigated, but associations with parental migration background remained after adjustment for education. Higher parental income was also associated with better cardiometabolic health but no longer when adjusted for parental education, which indicates a lesser impact on health. In addition, parental education modified the adverse associations between parental migration status and health outcomes such that they were only observed in children with low parental education. Taken together, these findings suggest that better health literacy in highly educated parents may be more important than high income, and that high education may compensate potential difficulties related to migration. In this sense, low education and parental migration background present a double disadvantage for the affected individuals’ health.

In our study, higher levels of maternal BMI and lower levels of sports club activity emerged as factors explaining the higher likelihood of poor cardiometabolic health associated with parental migration background. The positive association between maternal BMI and cardiometabolic risk factors is in line with previous research and is likely explained by genetic predisposition, intrauterine mechanisms related to pre-pregnancy maternal obesity, and shared environmental exposures such as obesogenic diet [34, 35]. Lack of physical activity is an important risk factor for childhood overweight, and several studies have found that migrant children have lower physical activity than native children [6, 36]. An explanation could be that migrant parents have less money to spend on club sport, are less familiar with local sport facilities and other opportunities for physical activity, or have different attitudes towards outdoor play [37]. According to previous studies, cultural differences in body image perception and body size preference may also promote different body weight in children of migrants compared to those of non-migrant parents [38, 39]. Both aspects encourage the implementation of lifestyle interventions for children as well as health education for children and parents that may effectively improve the health of immigrant children and their families.

The main strengths of this study are the European focus representing diverse regions with a variety of exposures, the availability of a large number of biological risk markers as well as behavioural and lifestyle indicators (albeit self-reported) and the high degree of methodological standardization across the eight countries. However, the cross-sectional study design is a limitation. While a longitudinal study of change in metabolic health between baseline and 6-year follow-up would have been preferable, sample size for observations of metabolic syndrome score would have been too small. Also, the limited sample size did not allow to estimate the extent to which maternal BMI and sports club activity explained the associations between parental migration background and health outcomes as the latter were no longer significant in the subsample with complete information on confounding variables. Furthermore, we did not distinguish migrant background based on country of origin (due to limited sample size) or reasons of migration (due to lack of information). Also, native parents may include second or third generation migrants who still experience disadvantages that distinguish them from the general population. We did not have any measures of acculturation, such as duration of residence or language ability, or information on whether the children had their own migration experience. Proxies for behavioural factors provide a limited picture of the children’s characteristics, and a finer categorization of the socioeconomic indicators would give a better picture of the impact of socioeconomic status. The small proportion of children with migrant background is a limitation, however, the study helps to provide an important insight into an area not well-researched. Finally, we note that the survey samples are not representative of the respective country, and the limited sample size may make it difficult to assess country-specific effects of migration background.

## Conclusions

The current study adds evidence for a higher risk of adverse cardiometabolic health in children and adolescents in Europe with parental migration background in comparison to their peers with native parents, with a differential impact of having one or two migrant parents. Excess body weight and clustering of other cardiometabolic risk factors in children is associated with higher morbidity and mortality in adulthood and underscores the need of action against the increasing prevalence of overweight and obesity in children. This seems to be particularly important for children with migration background, whose parents have had fewer educational opportunities.

### Supplementary information

Supplement

## Data Availability

The data that support the findings of this study are available from IDEFICS [http://www.idefics.eu] and the I.Family Study [http://www.ifamilystudy.eu] but restrictions apply to the availability of these data, which were used under license for the current study, and so are not publicly available. Data are however available from the authors upon reasonable request and with permission of the IDEFICS consortium.
