# Supplementary material for: Cardiometabolic risk profile among children with migrant parents and role of parental education: the IDEFICS/I.Family cohort
Source: Int J Obes (Lond). 2023 Sep 1;47(11):1074–80. doi: 10.1038/s41366-023-01359-5 (PMC10600002; doi:10.1038/s41366-023-01359-5)
Supplement: Supplementary file 1 — Supplement [file 41366_2023_1359_MOESM1_ESM.pdf]

Table S1. Human development index (HDI) for country of residence in relation to mean HDI for children with parental migration background (n = 8 745)

Country of residence	HDI	Largest migrant groups [a]	Mean HDI [b]	
			One migrant parent	Two migrant parents
Sweden	0.945	Near East [d] (30%)<br>Former Yugoslavia (13%)	0.907	0.765
Estonia	0.892	Former UDSSR [c] (54%)<br>Latvia, Lithuania (12%)<br>North America (10%)	0.868	0.881
Hungary	0.854	Romania (50%)<br>Former Yugoslavia (26%)	0.855	0.841
Germany	0.947	Near East [d] (41%)<br>Former UDSSR [c] (28%)<br>Poland (10%)	0.876	0.798
Belgium	0.931	Near East [d] (23%)<br>Germany (14%)<br>South and middle America (14%)	0.904	0.772
Spain	0.904	Africa (33%)<br>South and middle America (27%)<br>Far East (11%)	0.855	0.689
Italy	0.892	Switzerland (28%)<br>Germany (17%)<br>South and middle America (14%)	0.892	0.813
Cyprus	0.887	Former UDSSR [c] (28%)<br>Greece (22%)	0.862	0.819
Overall	*0.900*	*Former UDSSR [c] (20%)*<br>*Near East (19%)*	*0.876*	*0.797*

[a] *Most common countries of origin for migrant parents in the study population (≥ 10%)*

[b] *Mean parental HDI based on both parents' country of origin*

[c] *without Baltic States*

[d] *including Turkey*

Mean parental HDI differed across categories of parental migration background overall and within countries (Kruskal-Wallis test: $p < 0.0001$)

Table S2. Associations between parental migration background and cardiometabolic health indicators by parental education level [a]

		One migrant parent vs native parents		Two migrant parents vs native parents		Interaction p-value [b]
	N	Coefficient	CI 99%	Coefficient	CI 99%	
**BMI z-score**	8745					0.0004
Low education		**0.173**	**0.016, 0.330**	**0.385**	**0.199, 0.572**	
High education		-0.033	-0.195, 0.130	-0.007	-0.252, 0.238	
**MetS z-score**	3522					0.01
Low education		0.051	-0.146, 0.248	**0.471**	**0.214, 0.727**	
High education		-0.072	-0.276, 0.131	0.043	-0.262, 0.349	
**WC z-score**	8279					0.001
Low education		0.148	-0.064, 0.360	**0.494**	**0.240, 0.748**	
High education		-0.045	-0.265, 0.176	-0.064	-0.402, 0.275	
**SBP z-score**	7976					0.8
Low education		0.125	-0.020, 0.270	0.135	-0.043, 0.313	
High education		0.074	-0.076, 0.225	0.138	-0.094, 0.369	
**DBP z-score**	7975					0.6
Low education		0.041	-0.096, 0.178	0.158	-0.011, 0.326	
High education		0.001	-0.141, 0.143	0.055	-0.164, 0.274	
**HDL-C z-score**	4943					0.16
Low education		-0.067	-0.249, 0.116	**-0.255**	**-0.487, -0.023**	
High education		0.043	-0.145, 0.232	-0.019	-0.306, 0.269	
**TG z-score**	5031					0.5
Low education		0.054	-0.122, 0.231	**0.408**	**0.184, 0.633**	
High education		-0.005	-0.186, 0.176	0.261	-0.017, 0.539	
**HOMA-IR z-score**	3556					0.6
Low education		0.077	-0.161, 0.315	**0.316**	**0.008, 0.624**	
High education		-0.024	-0.271, 0.222	0.192	-0.201, 0.538	

[a] *Each model is adjusted for sex, age, country of residence; results indicated in bold are significant at 0.01 significance level*

[b] *P-value for a likelihood-ratio test comparing a model with two interaction terms with a model without interactions*

N, number of observations; CI, confidence intervals; BMI, body mass index; MetS, metabolic syndrome score; WC, waist circumference; SBP, systolic blood pressure; DBP, diastolic blood pressure; HDL-C, high-density lipoprotein cholesterol; TG, triglycerides; HOMA-IR, homeostasis model assessment for insulin resistance.

Table S3. Country-specific estimates for the associations between parental migration background and BMI z-score and metabolic syndrome z-score (ref = two native parents)[a]

	**One migrant parent vs native parents**		**Two migrant parents vs native parents**	
	Coefficient	CI 99%	Coefficient	CI 99%
**BMI z-score (n = 8745)** [b]				
Sweden	-0.088	-0.452, 0.275	-0.034	-0.479, 0.411
Estonia	0.144	-0.291, 0.580	0.100	-1.145, 1.344
Hungary	0.218	-0.317, 0.753	0.782	-0.405, 1.969
Germany	**0.323**	**0.004, 0.642**	**0.437**	**0.213, 0.662**
Belgium	0.128	-0.689, 0.946	0.591	-0.727, 1.909
Spain	-0.198	-0.841, 0.445	-0.143	-0.743, 0.457
Italy	0.080	-0.141, 0.300	-0.115	-0.666, 0.437
Cyprus	0.013	-0.181, 0.206	0.193	-0.107, 0.493
**MetS z-score (n = 3522)** [c]				
Sweden	0.043	-0.443, 0.528	-0.010	-0.662, 0.641
Estonia	-0.012	-0.492, 0.467	-0.047	-1.783, 1.689
Hungary	0.105	-0.717, 0.928	0.216	-1.526, 1.958
Germany	0.251	-0.169, 0.670	**0.632**	**0.334, 0.930**
Belgium	-0.248	-1.678, 1.183	0.577	-0.854, 2.001
Spain	-0.226	-1.312, 0.859	0.575	-1.171, 2.322
Italy	0.071	-0.191, 0.333	-0.029	-0.817, 0.750
Cyprus	-0.168	-0.405, 0.069	-0.016	-0.365, 0.333

[a] *Each model is adjusted for sex, age, country of residence, parental education; results indicated in bold are significant at 0.01 significance level*

[b] *Overall interaction p-value=0.09*

[c] *Overall interaction p-value=0.08*

N, Number of observations; CI, confidence intervals; BMI, body mass index; MetS, metabolic syndrome score

Table S4. Associations between parental migration background and cardiometabolic health indicators with and without adjustment for additional confounders in identical data sets [a]

		**One migrant parent vs native parents**		**Two migrant parents vs native parents**	
	N	Coefficient	99% CI	Coefficient	99% CI
**BMI z-score**					
Model 1	3 061	0.047	-0.145, 0.240	0.178	-0.146, 0.501
Model 2	3 061	0.050	-0.133, 0.233	0.150	-0.159, 0.459
**MetS z-score**					
Model 1	1 371	-0.027	-0.269, 0.215	0.113	-0.259, 0.484
Model 2	1 371	-0.015	-0.250, 0.221	0.090	-0.274, 0.455
**WC z-score**					
Model 1	2 965	-0.038	-0.297, 0.220	0.089	-0.343, 0.520
Model 2	2 965	-0.029	-0.277, 0.219	0.060	-0.356, 0.476
**SBP z-score**					
Model 1	2 918	0.175	-0.003, 0.352	0.279	-0.021, 0.578
Model 2	2 918	0.175	-0.002, 0.352	0.282	-0.018, 0.583
**DBP z-score**					
Model 1	2 917	0.045	-0.127, 0.218	0.232	-0.059, 0.523
Model 2	2 917	0.046	-0.126, 0.218	0.241	-0.051, 0.533
**HDL-C z-score**					
Model 1	1 915	0.031	-0.193, 0.255	0.157	-0.198, 0.512
Model 2	1 915	0.028	-0.195, 0.251	0.179	-0.178, 0.536
**TG z-score**					
Model 1	1 948	0.032	-0.184, 0.248	0.142	-0.200, 0.484
Model 2	1 948	0.038	-0.173, 0.252	0.143	-0.200, 0.486
**HOMA-IR z-score**					
Model 1	1 373	0.181	-0.132, 0.494	0.124	-0.357, 0.604
Model 2	1 373	0.185	-0.126, 0.496	0.094	-0.387, 0.575

[a] *Model 1: adjusted for sex, age, country of residence, parental education; Model 2: further adjusted for parental income, maternal BMI, sport club activity, fruit/vegetable intake; results indicated in bold are significant at 0.01 significance level*

N, Number of observations; CI, confidence intervals; BMI, body mass index; MetS score, metabolic syndrome score; WC, waist circumference; SBP, systolic blood pressure; DBP, diastolic blood pressure; HDL-C, high-density lipoprotein cholesterol; TG, triglycerides; HOMA-IR, homeostasis model assessment for insulin resistance.
